# Pushing the Survival Bar Higher: Two Decades of Innovation in Lung Transplantation

**DOI:** 10.3390/jcm13185516

**Published:** 2024-09-18

**Authors:** Khalil Aburahma, Nunzio Davide de Manna, Christian Kuehn, Jawad Salman, Mark Greer, Fabio Ius

**Affiliations:** 1 Department of Cardiothoracic, Transplant and Vascular Surgery, Hannover Medical School, 30625 Hannover, Germany; aburahma.khalil@mh-hannover.de (K.A.); demanna.nunzio@mh-hannover.de (N.D.d.M.); kuehn.christian@mh-hannover.de (C.K.); salman.jawad@mh-hannover.de (J.S.); 2 German Centre for Lung Research (DZL/BREATH), 35392 Hannover, Germany; greer.mark@mh-hannover.de; 3 Department of Respiratory Medicine and Infectious Diseases, Hannover Medical School, 30625 Hannover, Germany

**Keywords:** lung transplantation, survival, acute respiratory distress syndrome, ex-vivo lung perfusion, extracorporeal membrane oxygenation, antibody-mediated rejection

## Abstract

Survival after lung transplantation has significantly improved during the last two decades. The refinement of the already existing extracorporeal life support (ECLS) systems, such as extracorporeal membrane oxygenation (ECMO), and the introduction of new techniques for donor lung optimization, such as ex vivo lung perfusion (EVLP), have allowed the extension of transplant indication to patients with end-stage lung failure after acute respiratory distress syndrome (ARDS) and the expansion of the donor organ pool, due to the better evaluation and optimization of extended-criteria donor (ECD) lungs and of donors after circulatory death (DCD). The close monitoring of anti-HLA donor-specific antibodies (DSAs) has allowed the early recognition of pulmonary antibody-mediated rejection (AMR), which requires a completely different treatment and has a worse prognosis than acute cellular rejection (ACR). As such, the standardization of patient selection and post-transplant management has significantly contributed to this positive trend, especially at high-volume centers. This review focuses on lung transplantation after ARDS, on the role of EVLP in lung donor expansion, on ECMO as a principal cardiopulmonary support system in lung transplantation, and on the diagnosis and therapy of pulmonary AMR.

## 1. Introduction

Lung transplantation is the gold standard therapy for end-stage lung diseases that are refractory to more conservative therapies. However, survival after lung transplantation is worse than survival after other solid organ transplantations. According to the 36th adult and 22nd pediatric lung and heart-lung transplant reports of the International Society for Heart and Lung Transplantation (ISHLT), unadjusted 5-year survival amounted to 55.7% for adults and 53.1% for pediatric patients [1,2,3]. The survival of lung-transplanted patients has been impaired by the development of primary graft dysfunction (PGD), acute infections, malignancies, and acute and chronic lung allograft rejection (CLAD). CLAD includes two distinct phenotypes, the Bronchiolitis Obliterans Syndrome (BOS) and the more recently identified Restrictive Allograft Syndrome (RAS) [4,5]. According to the ISHLT registry reports, 5-year freedom from BOS amounted to only 50% and 45.7% in adult and pediatric patients, respectively [1,2,3,6].

**Figure 1:**
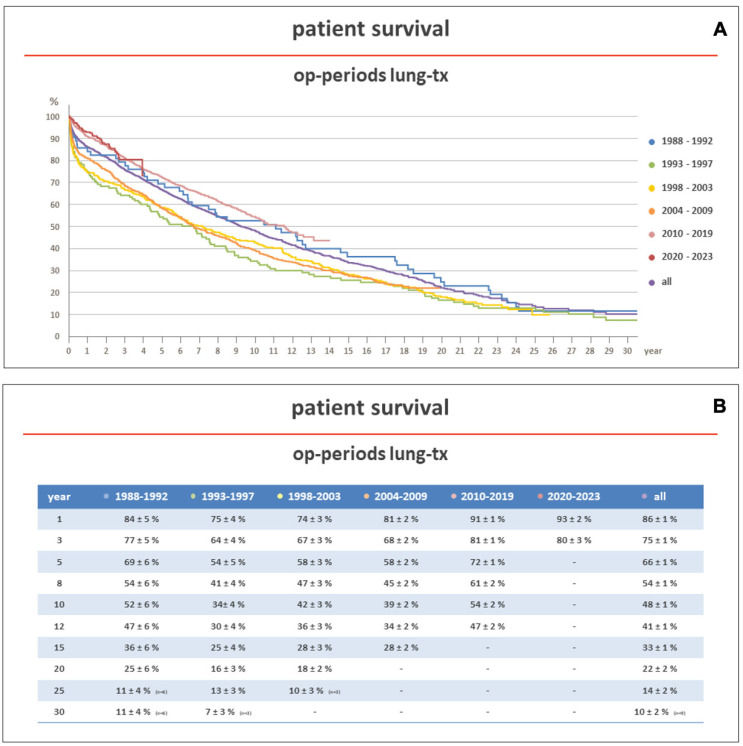
(**A**,**B**) show the survival of patients transplanted at the Hannover Medical School between 1983 and 2023, stratified according to the transplant era. Early survival was better in patients transplanted in more recent years (2010–2023). Op: operation; Tx: transplantation.

In more recent years (2010–2017), according to the same ISHLT reports, 5-year survival after lung transplantation has improved to 58.6% and 58.5% in adult and pediatric patients, respectively. This effect has been also observed at our institution, a center member of Eurotransplant (ET), where, between 1988 and 2023, 2639 patients underwent lung transplantation. The five-year unadjusted survival improved from 54% in the years between 1993 and 1997 to 72% in the years between 2010 and 2019 (Figure 1A,B).

This positive trend over the last two decades is explained in part by the great standardization of patient selection for lung transplantation and in part by the recent introduction and refinement of more versatile systems for extracorporeal life support (ECLS), such as extracorporeal membrane oxygenation (ECMO), for donor lung optimization, such as ex vivo lung perfusion (EVLP), and by the introduction of therapies for the treatment of acute cellular and antibody-mediated rejection (ACR and AMR, respectively), and for the prevention and treatment of viral, bacterial and fungal infections [7].

In this review, the contributors to this positive survival trend will be thoroughly discussed, with a particular focus on ECMO, EVLP and AMR.

## 2. Patient Selection and Indications

In lung transplantation, patient selection is of paramount importance and has been regulated by guidelines published by the ISHLT and the national general medical associations [8]. The recipient characteristics that were considered relative or absolute contraindications to transplantation two decades ago have now been accepted by most transplant centers. For example, older age (65–70 years) is not considered a contraindication to transplantation anymore, especially if patients have a low degree of frailty.

Donor organ allocation according to urgency and the post-transplant survival benefit are the most important rules for suggesting lung transplantation. Lung allocation has been regulated by urgency scores, such as the T (transplantable) and HU (high urgency) system, and the Lung Allocation Score (LAS) and its most recent update, the Composite Allocation Score (CAS) [9]. Each country has adopted its own allocation algorithm, and this may differently impact patient outcomes [10,11]. For example, Germany and The Netherlands use the LAS, while Austria and Belgium still use the T-HU system. In France, the super urgent status was introduced to prioritize very sick recipients with pulmonary fibrosis and primary pulmonary hypertension [11]. However, the ideal allocation score still does not exist [12,13,14]. In the US, the recently introduced CAS gave additional priority points to allosensitized patients, patients with the 0 blood group and small- or large-sized patients [15].

In adults, the most frequent indications for lung transplantation are end-stage lung emphysema and pulmonary fibrosis. Historically, cystic fibrosis-associated lung disease represented the third indication for lung transplantation. However, the introduction of triple channel modulator therapy has effectively reduced the indication for lung transplantation in patients with cystic fibrosis [16]. A similar trend can be expected in the future in patients with primary pulmonary hypertension (PPH) after the introduction of the activin signalling inhibitor sotatercept [17].

During the last decade, transplant indications have changed in pediatric (<18 years old) patients too, where the most common transplant indications have become PPH and childhood interstitial lung disease (CHILD); meanwhile, similar to adults, the transplant indication for cystic fibrosis has decreased [18]. This change has significantly influenced patient peritransplant management and has posed new operative challenges. In fact, more children aged < 12 years old are transplanted than before and such patients more often require peritransplant cardiopulmonary support than older children [18].

On the contrary, the number of transplants for other indications, such as acute end-stage lung disease after acute respiratory distress syndrome (ARDS), has increased in the last 5 years [19,20].

## 3. Transplant Indication for ARDS

Before the COVID-19 pandemic, lung transplantation was rarely indicated for acute end-stage lung disease after ARDS, due to the following reasons. First of all, there were no criteria for defining irreversible end-stage lung damage. To make it more complicated, there was no time threshold for defining irreversibility, as some patients with prolonged venous–venous ECMO runs recovered enough lung function to be weaned off it [21,22]. Second, many ARDS patients had obviously not undergone an evaluation for lung transplantation before ARDS occurred, and many were sedated and mechanically ventilated at the time of evaluation, thus being unable to consent to transplantation. Third, long-term post-transplant survival data were not available.

**Figure 2:**
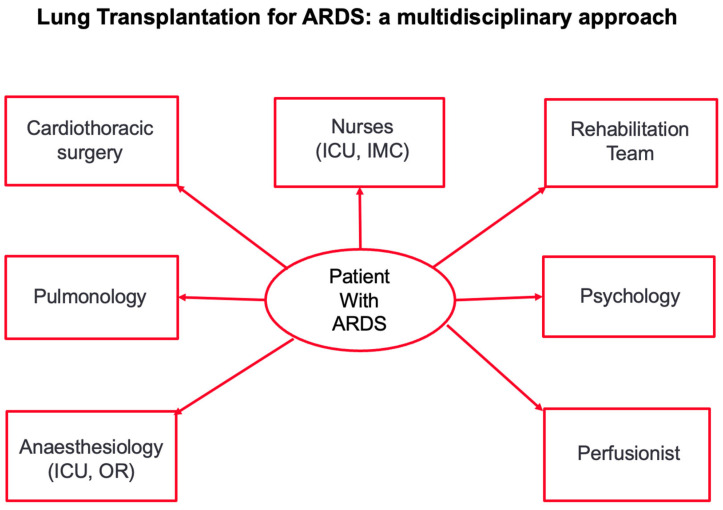
Figure 2 shows the multidisciplinary approach to transplantation in patients with irreversible lung damage after ARDS. ICU: intensive care unit; IMC: intermediate care station.

The COVID-19 pandemic encouraged the revision of multicentric experience to get more insights into lung transplantation for ARDS. In these patients, the survival results were encouraging, but they were usually obtained in highly selected patients [23,24,25,26]. Criteria for lung transplantation in patients with irreversible lung failure after ARDS have been recently published. The following “rules” were proposed [19,27]:

1. Patients should be younger than 65 years, since the results of ECMO as a bridge to transplantation (BTT) have been worse in older patients. However, not only chronological age but also patient frailty should be considered.
2. Patients should preferably only have single-organ dysfunction. However, patients requiring temporary hemodialysis have been successfully transplanted too. Concomitant liver dysfunction or left heart failure should be considered a contraindication to lung transplantation. The development of right heart failure due to secondary pulmonary hypertension should not be considered a contraindication to transplantation. In this case, veno-venous (v-v) ECMO should be upgraded to veno-arterial (v-a) or veno-arterial-venous (v-a-v) ECMO.
3. Enough time should be allowed for lung recovery, usually 4–6 weeks after the initial event. Evaluation for lung transplantation may be considered earlier than 4 weeks, in case of potentially lethal pulmonary complications that cannot be managed medically or through the use of ECMO.
4. There should be radiological evidence of irreversible lung disease, such as extensive honeycombing, cystic changes, reticular opacities, and traction bronchiectasis, >80% lung involvement, and a right atrium to left atrium ratio > 1; these are associated with an extended period of static respiratory mechanics or ECMO parameters [19]. The presence of secondary pulmonary arterial hypertension should be also considered a sign of irreversibility.
5. Patients should be awake and able to discuss transplantation. The advantages of “awake” ECMO as a bridge to lung transplantation are well known. However, in some patients, who are otherwise good candidates for transplantation, spontaneous breathing is not possible. In this case, a patient’s next-of-kin may consent to transplantation.
6. Patients should be able to participate in physical rehabilitation while on the transplantation waiting list.
7. Patients should fulfil the remaining typical criteria for transplantation; these include, for example, an adequate body mass index and an absence of other notable conditions, such as severe coronary artery disease or a history of active smoking.
8. Patients should have a negative SARS-CoV-2 virology status.
9. Transplantation for ARDS should be performed only in high-volume centers that have substantial experience with ECMO as a bridge to transplantation.
10. The transplant center should have access to a broad donor pool and low waiting list mortality. The clinical condition of patients with irreversible lung failure after ARDS may rapidly worsen, thus precluding transplantation. Continuous patient re-evaluation is therefore needed.
11. These criteria and the indication for transplantation should be evaluated, discussed and approved by the multidisciplinary transplant conference. A multidisciplinary approach is of paramount importance in successfully transplanting patients after irreversible lung damage due to ARDS (Figure 2).

In the last 3 years, several case series on lung transplantation in highly selected patients after COVID-19 ARDS have been published [28,29,30,31,32,33]. In 2021, Bharat et al. published the first multicentric experience with lung transplantation in 12 patients with COVID-19 ARDS. All patients were on mechanical ventilation (MV, median time, 70 days; interquartile range, IQR, 49–80 days) and 11 patients were on ECMO (median time, 55 days; IQR, 44–79 days) before transplantation. After a waiting time of 6 days, the patients were transplanted using a clamshell approach and switching v-v to veno-arterial (v-a) ECMO. All the explanted lungs showed massive fibrosis. After transplantation, the median MV time was 16 (4–21) days, the median intensive care unit (ICU) time was 20 (13–24) days, and the median hospital stay was 37 (27–42) days; 30-day survival was 100% [28].

In a study based on the United Network for Organ Sharing (UNOS) database, Roach et al. showed that, between August 2020 and September 2021, 214 (7.0%) transplantations were performed for COVID-19-related respiratory failure (65% after ARDS, 35% for pulmonary fibrosis after COVID-19 ARDS). The 30-day mortality was 2.2%, and the 3-month survival was 95.6% [29].

In 19 (18%) out of 106 patients referred for transplantation between January 2020 and May 2021, Lang et al. reported a median (IQR) time on ECMO as a bridge to transplantation (BTT) of 41 (2–66) days. All patients underwent bilateral lung transplantation on v-a ECMO support. The median MV time was 24 (4–82) days, the median ICU time was 36 (12–98) days, and the median hospital stay was 64 (47–450) days; the 30-day survival was 100%. The 3-month survival did not differ between patients transplanted for COVID-19 ARDS and patients transplanted for other indications [30].

A propensity-matched analysis of 11 patients transplanted for COVID-19 and 163 hospitalized patients transplanted for other indications demonstrated comparable rates of intrathoracic adhesions, a comparable posttransplant duration of MV, PGD 3 at 72 h, and delayed chest closure between groups. However, patients transplanted for COVID-19 ARDS had significantly longer posttransplant hospital stays (48 vs. 23.5 days), a higher prevalence of acute rejection ≥ A2 (40% vs. 0%) and donor-specific antibodies > 2000 MFI (70% vs. 0%), but comparable 1-year survival rates [31].

Another analysis from the UNOS database showed that the 6-month survival did not differ between matched patients (*n* = 227) transplanted for COVID-19 respiratory failure and patients (*n* = 454) transplanted for other lung diseases (94.4% vs. 88.1%, *p* = 0.26) [32].

Finally, Bermudez et al. showed that the 1-year survival was similar between 188 patients transplanted for COVID-19 ARDS and 117 patients transplanted for post-COVID pulmonary fibrosis (87.3% vs. 86.7%). Among these patients, the 1-year survival did not differ between patients who required pretransplant ECMO and patients who did not require it (84.8% vs. 90.9%, *p* = 0.2). Additionally, the 1-year survival was similar in recipients requiring ECMO for COVID-19 lung failure and recipients requiring ECMO for non-COVID restrictive lung failure (84.8% vs. 78.0%, *p* = 0.1) [33].

## 4. Donor Optimization and EVLP

Standard cold preservation is the gold standard for donor lung preservation. However, once the donor lungs have been perfused and stored in the transport box, no other intervention or evaluation is possible, and the cold ischemic time is running until organ reperfusion. Thus, one of the first rules in standard cold preservation is to keep the ischemic time as short as possible to avoid posttransplant PGD [34,35,36].

First applied in 2001, ex vivo lung perfusion (EVLP) represents one of the most important recent innovations in lung transplantation [37,38,39,40,41,42,43]. The technique of EVLP comprises the perfusion of donor organs outside the body and has been developed to preserve explanted donor lungs in a perfused, ventilated and normothermic condition, thus decreasing tissue ischemia and lung inflammation, while allowing for extended evaluation and potential reconditioning prior to transplantation.

**Figure 3:**
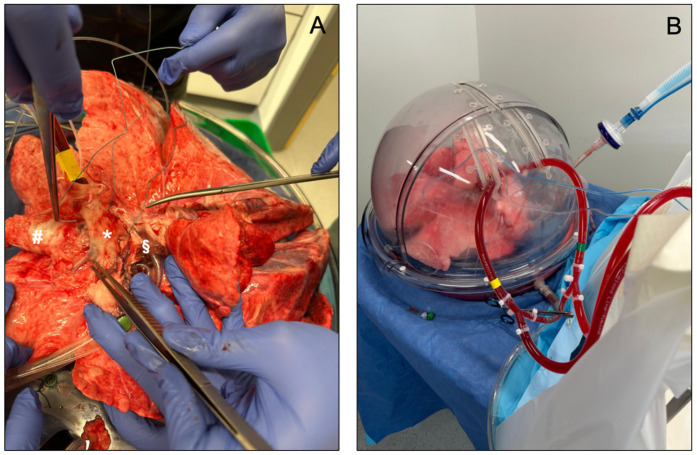
(**A**,**B**) show an EVLP set-up using the XPS XVIVO Perfusion AB system (XVIVO Perfusion, Gothenburg, Sweden). In (**A**), * identifies the pulmonary artery, # the trachea, and § the left atrium.

To date, three distinct EVLP devices have been approved for clinical use: the Organ Care System Lung (OCS^TM^ Lung, TransMedics, Andover, MA, USA), the Vivoline LS1 system (Vivoline Medical, Lund, Sweden) and the XPS XVIVO Perfusion AB system (XVIVO Perfusion, Gothenburg, Sweden, Figure 3). The three systems have a common set-up, including an organ chamber, a reservoir for collecting the perfusion fluid, a pump (roller, centrifugal, or piston-driven), a ventilator, and an oxygenator. Interventions such as bronchoscopy are possible in all three systems.

However, in clinical application, the EVLP systems differ in some of their technical characteristics and perfusate properties (Table 1). The OCS^TM^ system uses OCS^TM^ solution with red blood cells (RBCs, cellular perfusate) to target a hematocrit between 15% and 25%, an open left atrium (LA) and a pulmonary artery flow of 2–3 L/min. The Vivoline LS1 system uses the Steen solution added with RBCs to target a hematocrit between 10% and 15% (the so-called Lund protocol). The LA is left open. A pulmonary arterial flow of 100% cardiac output (CO) is used. The XPS system utilizes Steen solution but without RBCs (acellular perfusate, the so-called Toronto protocol). The LA is left closed by suturing the LA draining cannula, so that the LA pressure reaches 3 to 5 mmHg. The pulmonary artery flow is kept at 40% of CO.

The OCS^TM^ Lung is the only EVLP portable system, meaning that EVLP is initiated at the donor hospital and, thus, the cold ischemic time is minimized. The other two EVLP systems are static, implying that EVLP initiation occurs at the recipient hospital and, therefore, that the cold ischemic time is significatively longer than the cold ischemic time in the OCS-driven EVLP runs.

The EVLP benefits/added values are the following: (1) a reduction in the cold ischemic time and the risk of ischemia–reperfusion injury (IRI), which clinically manifests as PGD after transplantation. PGD is a well-known risk factor for the development of CLAD [44]; (2) the expansion of the lung donor pool, by allowing the evaluation and reconditioning of extended-criteria (marginal) donors (ECD), which represent 40% of the donor pool, and of lungs from donors after circulatory death (DCD). This last option is not available in Germany, where, in 2022, only 242 of the 459 (53%) offered donor lungs were finally transplanted; (3) the application of therapies during EVLP. In fact, antibiotic treatment in donor lungs with known bacterial infections, antiviral treatment using ultraviolet irradiation in lungs from HCV-infected donors, and thrombolysis in lungs from donors who died of acute pulmonary embolism can be safely started and performed while the lungs are being perfused; and (4) it is an ideal research platform to investigate new treatments (translational research from bench to the bedside), such as cell-based therapies with the infusion of mesenchymal stromal cells and regulatory T cells, anti-inflammatory therapies using alpha1 antitrypsin, and the lavage of donor lungs after aspiration using surfactant [38,39,41,43].

Clinical studies performed during the last 13 years have shown the non-inferiority of EVLP in comparison to standard cold preservation [45,46,47,48]. A single-center, randomized controlled study, including 80 patients, demonstrated a lower % of PGD > 1 at all time points in patients whose donor lungs were perfused using EVLP, but it did not reach statistical significance due to an exiguous PGD Grade 3 prevalence in the control group [47]. The INSPIRE multicenter, randomized controlled study showed a decreased % PGD grade 3 at 72 h after transplantation and a tendency for shorter hospitalization in patients whose lung grafts were preserved using the OCS^TM^ Lung. Those effects did not translate into a beneficial effect on the 12-month survival altogether [48].

Other studies showed the EVLP potential in expanding the use of ECD lungs, which had not otherwise been accepted for transplantation [49,50,51,52]. In one of the first published studies on EVLP, the Toronto group was able to transplant 20 out of 23 (87%) high-risk donor lungs, which showed a lower prevalence of PGD than non-ECD lungs (15% vs. 30%) [49]. In the EXPAND multicenter, single-arm, pivotal trial, 87% of the OCS-perfused ECD lungs were successfully transplanted, with a 91% patient survival at 1 year, notwithstanding there being 44% of PGD grade 3 at 72 h after transplantation [51]. On the contrary, in the DEVELOP-UK multicenter, observational study, only 18 out of 53 (34%) ECD lungs were successfully transplanted after EVLP perfusion using the Lund protocol. Patients transplanted with ECD lungs showed a more complicated posttransplant course than patients transplanted with standard lungs [50].

Notwithstanding the versatility and potential benefits of EVLP, a United Network Organ Sharing (UNOS) survey showed that only 4.7–6.5% of lung transplantations were performed using EVLP [39]. Moreover, the results after EVLP use vary according to the transplant center volume and experience [53,54]. Logistics and EVLP costs are of major concern, impeding the widespread use of EVLP, such as in Germany, where the EVLP costs are still not covered by health insurance. However, recent studies from the US have demonstrated the cost effectiveness of EVLP in lung transplantation [55,56].

Recently, our colleagues in Toronto investigated the role of static lung storage at 10 °C instead of the standard 4 °C for prolonging the graft cold ischemic time. The rationale behind this new cold storage strategy lies in the better mitochondrial protection achieved at 10 °C than 4 °C. In 2021, Ali et al. presented their first experience with prolonged lung graft preservation at 10 °C in five patients. The median preservation time amounted to 10.4 h for the first implanted lung and 12.1 h for the second implanted lung. All patients survived without any PGD grade 3 at 72 h after transplantation [57]. The authors confirmed these results in a prospective, multicenter, nonrandomized trial, comparing outcomes between 70 patients, whose grafts were preserved at 10 °C, and 140 matched controls. For the 70 case patients, the lung grafts were retrieved and transported to the recipient hospital at 4 °C and then preserved at 10 °C in a specific incubator for a median of 7.48 h. Thus, the median ischemic time for the first implanted lung amounted to 12.28 h, and amount to 14.08 h for the second implanted lung. The prevalence of PGD grade 3 at 72 h was 5.7% in the study group and 9.3% in the control group. The need for postoperative ECMO support, a stay in the ICU and hospital, and the 1-year survival did not differ between groups [58].

A new portable lung preservation device has been recently introduced for controlled hypothermic storage (CHS); this is the LUNGguard (Paragonix Technologies, Waltham, MA, USA), which allows CHS at 4–8 °C. In a European prospective multicenter observational study, Provoost et al. used the LUNGguard for CHS in 36 patients undergoing lung transplantation. The CHS temperature was 6.5 °C (3.7–9.3 °C) and the ischemic times were 13.38 h and 15.41 h for the first and second implanted lung. The prevalence of PGD grade 3 at 72 h amounted to 2.8%. Only one patient died in the hospital [59].

By safely prolonging the cold ischemic time, CHS may improve lung transplantation logistics and performance, allowing day-time transplantation [58,59].

## 5. ECLS or the Holy Grail in Lung Transplantation

In many patients, lung transplantation is possible without the use of extracorporeal life support (ECLS) [60].

Among the available ECLS systems, ECMO is by far the most employed in lung transplantation. Moreover, in comparison to heart transplantation, there is still no long-term bridging assist device in lung transplantation. Extracorporeal carbon dioxide removal (ECOO_2_R) allows only partial decarboxylation. The central or peripheral Novalung (Novalung GmbH, Hechingen, Germany) system requires a full sternotomy or the cannulation of the femoral vessels, respectively, and depend on the cardiac function of the patient for pumping blood through the system [61,62,63,64,65,66,67].

On the contrary, ECMO versatility allows for pre-transplant support, which can be directly continued intraoperatively and post-transplant. The paramount role of ECMO in lung transplantation has been confirmed by numerous retrospective studies, without any randomized trial available altogether [60].

ECMO has been successfully used as BTT, especially in its veno-venous (v-v) configuration that may be upgraded to a veno-arterial venous (v-a-v) configuration in patients developing right heart failure or hemodynamic instability before transplantation, by implanting an additional arterial cannula in a femoral artery. The survival results of ECMO as a BTT have been improving in recent years due to better patient selection, the introduction of awake ECMO protocols and an increase in center transplant volume and experience, as aforementioned for lung transplantation in patients with ARDS [68,69,70,71,72,73,74,75,76,77,78,79,80,81,82,83,84,85,86,87]. In 2008, the “awake” strategy was introduced in the management of pre-transplant patients at our institution [73]. Since then, many other case series have validated the benefit of spontaneous breathing and mobilization during ECMO support, thus reducing patient muscular deconditioning due to immobilization and mechanical ventilation [68,69,70,71,78,81,85,88,89]. Moreover, patient rehabilitation during ECMO support has been improved by the introduction of new cannulas and implant techniques [90,91,92,93]. In our experience, the overall graft survival did not differ between BTT and non-BTT patients (79% vs. 90% and 61% vs. 68% at 1 and 5 years, respectively, *p* = 0.13) and ECMO as BTT was not a risk factor for mortality at the multivariate analysis [70]. Finally, another ECMO configuration that has recently been introduced for supporting right heart function and may have a promising role in lung transplant candidates as a BTT when v-v ECMO fails is right atrium (RA) to pulmonary artery (PA) bypass (RA-PA bypass). This system uses an ECMO circuit connected to a dual site single-lumen ProtekDuo Cannula (ProtekDuo^TM^ Kit, LivaNova, Munich, Germany) that is implanted percutaneously through the right internal jugular vein and advanced under radiologic or echocardiographic control until the PA. This system allows early patient mobilization and combines respiratory and hemodynamic support [94,95].

In many transplant centers like ours, starting from the early 2010s, ECMO in its veno-arterial configuration has replaced the use of cardiopulmonary bypass (CPB) for intraoperative support. Intraoperative ECMO allows for continuing support in patients who may not be weaned from it and the possibility of performing major cardiac surgery; for example, coronary artery bypass grafting (CABG) requires a lower amount of heparin and priming volume in comparison to CPB and causes a lower degree of systemic inflammatory reaction (SIRS) than CPB [96,97,98,99,100,101,102,103,104,105]. In two recent publications, our group has demonstrated that survival does not differ between patients who require intraoperative ECMO and those who do not, and that the intraoperative use of ECMO does not emerge as a risk factor for in-hospital mortality or mortality after hospital discharge [106,107]. Moreover, our group has developed a decision algorithm that helps in identifying those patients at risk of requiring intraoperative ECMO support in order to avoid ECMO implant under urgent/emergent conditions [106].

Coronary artery bypass grafting (CABG) can be performed safely under v-a ECMO support, with or without cardioplegic arrest. We have recently demonstrated that this procedure is feasible, and that patients undergoing concomitant CABG and lung transplantation show survival equivalent to that of patients without severe coronary artery disease (*p* = 0.556). At our institution, CABG is usually performed directly after lung transplantation [108].

CPB still has a limited role in lung transplantation, particularly in patients requiring concomitant cardiac surgical procedures other than CABG, in small infants, and in patients where unexpected massive blood loss due to injury of the pulmonary artery or of the left atrium occurs during intraoperative dissection [106,107].

After transplantation, ECMO has an important role in rescuing grafts that develop severe graft dysfunction due to PGD, acute rejection and pneumonia, refractory to more conservative therapies. However, graft function and survival are worse in patients who require secondary ECMO [109,110,111,112,113,114,115,116,117]. Thus, the early recognition of incoming graft dysfunction with prompt ECMO implant has improved graft prognosis [109]. In patients with PPH, the postoperative direct extension of intraoperative ECMO support has effectively reduced the prevalence of PGD [88,89,118]. In these patients, we and our colleagues in Vienna have recently demonstrated that the primary mechanism causing PGD might be more likely related due to a diastolic dysfunction of the left ventricle. In fact, the long-standing underfilling of the left ventricle in the presence of reduced cardiac output secondary to very high pulmonary vascular resistance may result in left ventricular deconditioning, rendering the left ventricle incapable of handling a normal preload during the early postoperative period. The characteristic pulmonary oedema of PGD is a consequence of a sudden increase in the left ventricular end diastolic pressure and in the left atrial pressures soon after graft reperfusion, aggravated further after weaning from mechanical ventilation. Postoperative v-a ECMO use provides time for gradual adaption of the left ventricle to the new hemodynamic situation after transplantation [88].

## 6. Antibody-Mediated Rejection in Lung Transplantation

Antibody-mediated rejection (AMR) has emerged as a relevant clinical and prognostic complication later in lung transplantation than in other solid organ transplantations, such as kidney transplantation [119,120,121,122,123]. Consequently, its definition, monitoring and treatment have been literally borrowed from kidney transplantation. However, in comparison to acute cellular rejection (ACR) [124,125], the definition of AMR in lung transplantation is still debated [126,127,128,129].

The development of anti-HLA DSAs after transplantation, also defined as allosensitization or alloimmunisation, is a major risk factor for AMR. Among the mismatched antigens between the donor and recipient, the HLA antigens play an important role in the processing and recognition of self vs. non-self antigens. Class I HLA antigens are expressed on the surface of all nucleated cells, and present foreign antigens to cytotoxic T lymphocytes. Contrarily, class II HLA antigens are expressed predominantly on the surface of antigen-presenting cells (APC) [130]. Recently, antibodies against non-HLA antigens (non-HLA DSAs) have been also associated with AMR and CLAD, acting synergically with anti-HLA DSAs [131,132,133]. The mechanisms of allograft injury and the treatment of anti-HLA and non-HLA DSAs are similar [134,135,136].

Anti-HLA DSAs are antibodies of IgG class (IgG1, IgG2, IgG3, and IgG4). The IgG1 and IgG3 classes bind the complement, but, at a high titer, all IgG classes may acquire this characteristic. DSAs are pre-formed, i.e., they exist before lung transplantation, if the recipient has already been exposed to HLA antigens; this is as a consequence of blood transfusions, previous lung transplantation and major surgery, and pregnancies. Pre-formed DSA are of prognostic importance, because they can cause hyperacute rejection. Therefore, every transplant recipient undergoes a screening for anti-HLA antibodies before listing. Moreover, a crossmatch, which is obtained by mixing donor lymphocytes and recipient serum, is performed before (prospective crossmatch) or soon after (retrospective crossmatch) transplantation. Since donor lungs are not offered to recipients that show antibodies against donor HLA antigens (forbidden antigens), highly sensitized patients face a longer waiting list time and increased waiting list mortality [136,137,138,139,140]. DSAs developing after lung transplantation are called de-novo DSAs. Patients may develop de-novo DSAs early, during the first weeks after transplantation, or later, usually just before or concomitantly with CLAD development.

The increase in DSA prevalence has paralleled the use of the more specific and sensitive Luminex assay [141]. In retrospective case series published over the last ten years [131,142,143,144,145,146,147,148,149,150,151,152,153,154,155,156,157,158,159,160,161,162,163,164,165], the prevalence of DSAs, either preformed or de novo, ranges between 4.3% in the study of Witt et al. [149], where only patients with AMR were considered, and 64.1% in the study of Roux et al. [155], yielding an overall a mean prevalence of 30.4%. DSAs were more often antibodies against class II HLA antigens, especially against the DQ antigens. Two studies showed a prevalence of complement-binding DSAs, at 42.9% and 39.7% [142,143,166,167]. The median time to DSA detection ranged from as early as 14 and 30 days after transplantation in the studies published by Le Pavec et al. [158] and Ius et al. [157] to a median of 537 days in the study published by Kim et al. [151]. Moreover, a failure to detects DSAs in the serum of patients with presumed AMR does not mean that DSAs are not produced at all, as they may be bound to the graft (intragraft DSA) [156,168,169].

In the prospective HLA antibodies after lung transplantation (HALT) study, 43 out of the 119 (36%) included patients showed DSAs after transplantation, being against class I HLA antigens in 14% of patients, against class II HLA antigens in 53% of patients, and against both HLA classes in 33% of patients. DSA specificity was most frequent for HLA-DQ antigens. DSAs were detected within the first 30 days of transplantation in 60% of patients, and thereafter in the remaining 40%. DSAs were pre-formed in 10% of patients [165].

In comparison to acute cellular rejection (ACR) [124,125], the definition of AMR in lung transplantation is debated and derives from the Banff definition of AMR in kidney transplantation [127,128,129]. In 2016, the ISHLT issued a consensus statement for the diagnosis of AMR [126]. The diagnostic criteria to be fulfilled were the following: the presence of DSAs; the presence of graft dysfunction; the exclusion of other causes of graft dysfunction, such as infection; the presence of a specific histology during the transbronchial biopsies, such as capillary neutrophilic infiltration and endotheliitis, diffuse alveolar damage (DAD), or the presence of acute fibrinous and organizing pneumonia (AFOP); and the presence of C4d deposits during the immunohistochemistry. Graft dysfunction distinguishes between the presence of clinical and subclinical AMR. The diagnosis of clinical AMR may be definite if all the previous criteria are present; probable if at least four criteria are fulfilled; and possible if at least three criteria are present. Similarly, the diagnosis of subclinical AMR may be definite if all criteria (DSA, histology and C4d) are present; probable if at least two criteria are fulfilled; and possible if at least one criterion is present [126].

These guidelines represent a significant improvement in the diagnosis of AMR in lung transplantation. However, this classification shows several limitations and a new consensus statement is going to be published by ISHLT in the near future. According to the authors of the ISHLT consensus statement, the main challenges in the diagnosis and grading of AMR in lung transplantation are the lack of specific diagnostic features and the variable relationship between DSA and the presence of graft damage and dysfunction. Therefore, a secure diagnosis of AMR mandates a multidisciplinary approach that integrates the clinical presentation with available immunologic and pathologic diagnostic tools [126]. The histologic criteria and the C4d deposition are the Achilles’ heel of this classification, because the histopathologic findings in AMR are non-specific patterns of injury that can be seen in a variety of disorders, including severe acute cellular rejection, infection (especially bacterial and viral), graft preservation injury and drug reactions [170]. Moreover, it is not always possible to evaluate all proposed criteria. For example, transbronchial biopsies may be a high-risk procedure in patients with severe graft dysfunction who would instead benefit from prompt therapy against AMR. This classification does not also clearly define the severity of AMR, and criteria for defining chronic AMR still have to be developed. Finally, other parameters may be considered in the future to help with the diagnosis of AMR, such as the detection of intragraft DSAs and of donor-derived cell-free DNA [171,172].

The development of DSAs and AMR have been associated with worse survival and an increased risk of CLAD. However, most of the evidence is based on retrospective, single-center studies [142,143,144,146,147,148,149,150,151,152,153,154,155,156,157,159,160,161,162,163,173,174]. The complement binding capacity, DSA persistency, anti-HLA DQ specificity, high MFI values and antibody titers, and evidence of intragraft DSAs have been invariably associated with mortality and CLAD. The knowledge of the mechanisms through which DSA provoke graft damage is of paramount importance for planning treatment and developing new drugs [175,176,177]. First of all, complement-binding DSAs activate the classical complement cascade. This reaction is initiated by the binding of DSAs with the complement factor C1q, and causes cellular damage directly through the formation of the membrane attack complex, and indirectly through the production of the anaphylatoxins C3a and C5a; in turn, these increase vascular permeability, the expression of adhesion molecules and inflammatory cell infiltration. DSAs also mediate antibody-dependent cell-mediated cytotoxicity (ADCC). Upon the binding of DSAs with the corresponding HLA antigens, the antigen/antibody complex is recognized by effector cells, such as natural killer (NK) cells, cytotoxic effector T cells, macrophages and neutrophilic granulocytes via the Fcγ receptor (FcγR). As a consequence, cell lysis ensues. The DSA-induced cross-linking of HLA class I and class II antigens on the cell surface, for example, on the surface of endothelial cells, activates the expression of leukocyte adhesion molecules and chemokines, which allows the infiltration of neutrophilic granulocytes and monocytes, thus starting graft damage.

Over the last two decades, several new drugs and interventions have been developed to treat DSAs and AMR in lung transplantation [136,175,176,177,178,179,180,181,182,183]. The available drugs target specific mechanisms of DSA production, such as Belatacept (Bristol-Myers Squibb GmbH, München, Germany), which blocks the CTLA4 costimulatory pathway in the germinal center; Tocilizumab (Roche Pharma AG, Grenzach-Wyhlen, Germany), an antagonist of the IL-6 receptor [184,185,186,187,188,189]; Clazakizumab (CSLBehring, Marburg, Germany), an antagonist of IL-6; Rituximab (Roche Pharma AG, Grenzach-Wyhlen, Germany), an anti-CD20 chimeric monoclonal antibody that targets B-cell production [190]; and the proteasome inhibitors Bortezomib (Ratiopharm GmbH, Ulm, Germany) and Carfilzomib (AMGEN GmbH, München, Germany) [191,192,193], and the anti-CD38 Daratumumab (Janssen-Cilag International NV, Beerse, Belgium), which inhibit DSA production by plasma cells. Other drugs target the mechanisms yielding to graft damage by inhibiting the complement cascade, such as Eculizumab (Alexion Europe SAS, Levallois-Perret, France), an inhibitor of the complement factor C5 [194,195,196], and Berinert (CSL Behring GmbH, Marburg, Germany), a C1 esterase inhibitor.

Extracorporeal photopheresis [197,198], therapeutic plasmapheresis (tPE) [199,200], immunoabsorption and Imlifidase (IdeS), a recombinant cysteine protease derived from *Streptococcus pyogenes* that rapidly cleaves IgG [201], remove DSAs from the bloodstream.

While the previous drugs and interventions act at a precise point of the immunologic cascade yielding to DSA production and graft damage, the human intravenous immunoglobulins (IVIG), which may contain only IgG or be enriched with IgA and IgM immunoglobulins (IgGAM, Pentaglobin, Biotest AG, Dreieich, Germany), have pleiotropic effects. They neutralize DSAs in the periphery before antigen recognition, scavenge activated complement, inhibit the activation of ADCC effector cells by FcγR blocking and the tissue migration of activated neutrophilic granulocytes and monocytes, and activate T regulatory cells [202,203,204,205,206,207,208,209,210,211,212,213,214].

The complexity of the immunologic cascade yielding to DSA production and AMR in lung transplantation and in other solid organ transplantations requires the treatment protocols to preferably include more than one drug or intervention, such as several sessions of tPE and immunoabsorption, IVIG, and Rituximab. This is particularly important in patients with preformed DSA who are at a high risk of hyperacute rejection if they were deliberately transplanted with a positive virtual crossmatch [215,216,217,218,219,220].

The treatment of de novo DSAs has been suggested only for patients who concomitantly show graft dysfunction [174,221]. However, the treatment of clinical AMR has shown disappointing results [148,149,222,223,224,225], especially considering that the diagnosis of clinical AMR is complex and time-consuming. Therefore, some authors have recently proposed that DSA treatment should be performed pre-emptively, before graft dysfunction ensues, as in patients with subclinical AMR [146,164].

## 7. Conclusions and Future Directions

Over the last two decades, the introduction of new techniques and therapies and the refinement of those already existing have significantly improved survival in lung transplantation. At our institution (Figure 1), the 5-year unadjusted survival of lung-transplanted recipients has now reached the 5-year survival of other solid organ transplant recipients, as in heart transplantation, where the 5-year unadjusted survival is 72.2% [226].

There is still room to push the survival bar further higher. The development of new primary immunosuppressive drugs other than the available calcineurin and m-TOR inhibitors, as well as cell-cycle inhibitors, is highly needed, with special attention paid to reducing the adverse side effects of drugs. The use of DCD donors, which is unfortunately not allowed in Germany, may reduce the gap between donor and recipient numbers, thus reducing waiting list mortality [227,228]. Transplantation across the HLA barrier may also reduce the waiting list mortality [215,216,217,218,219,220]. The availability of new therapies against AMR may open the door to ABO-incompatible lung transplantation, which shares many immunologic mechanisms with AMR itself [229,230]. Finally, the introduction of cell-based therapies, such as those based on regulatory T cells (Tregs), may improve graft tolerance, thus reducing the incidence of CLAD and immunosuppressive drug toxicity [231].

## Figures and Tables

**Table 1:** Table 1 shows the differences between the actually used EVLP protocols.

Parameter	Lund	OCS	Toronto
Perfusion			
Perfusate	Cellular	Cellular	Acellular
Solution	STEEN	OCS	STEEN
Cellular component	RBCs	RBCs	none
Target flow	100% cardiac output	2–2.5 L/min	40% cardiac output
Flow type	Continuous	Pulsatile	Continuous
PA pressure	<20 mmHg	<20 mmHg	<15 mmHg
Left atrium	Open	Open	Closed
			
Ventilation			
Start temperature	32 °C	34 °C	32 °C
Tidal volume	5–7 mL/kg	6 mL/kg	7 mL/kg
Respiratory rate	20/min	10/min	7/min
FiO_2_ (%)	0.5	0.12	0.21
			
Transportability	Static	Portable	Static

OCS: Organ Care System; PA: pulmonary artery; RBCs: red blood cells.

## Data Availability

The survival data reported in Figure 1 are available upon request.

## List of Abbreviations

ARDS	acute respiratory distress syndrome
BOS	bronchiolitis obliterans syndrome
BTT	bridge to transplantation
CABG	coronary artery bypass grafting
CBP	cardiopulmonary bypass
CLAD	chronic lung allograft dysfunction
CO	cardiac output
DCD	donation after circulatory death
eDSA	early donor-specific anti-HLA antibodies
ECD	extended-criteria donor
ECLS	extracorporeal life support
ECMO	extracorporeal membrane oxygenation
ET	Eurotransplant
EVLP	ex vivo lung perfusion
ISHLT	International Society of Heart and Lung Transplantation
LAS	lung allocation score
MFI	mean fluorescence intensity
PGD	primary graft dysfunction
RBCs	red blood cells
RAS	restrictive allograft syndrome
